# Study of Menstrual Patterns, Abnormalities, and Irregularities in Students

**DOI:** 10.7759/cureus.40206

**Published:** 2023-06-10

**Authors:** Hetal Rathod, Shreya Rathi, Shashank Tiwari, Chaitali Borgaonkar

**Affiliations:** 1 Community Medicine, Dr. D. Y. Patil Medical College, Hospital and Research Centre, Dr. D. Y. Patil Vidyapeeth, Pune, IND; 2 Medicine, Dr. D. Y. Patil Medical College, Hospital and Research Centre, Dr. D. Y. Patil Vidyapeeth, Pune, IND

**Keywords:** premenstrual symptoms, junk food, bmi, menstrual irregularities, dysmenorrhoea

## Abstract

Introduction

One-third to one-half of females with primary dysmenorrhea are missing school or work at least once per cycle, and more frequently 5% to 14% of them. Dysmenorrhea is one of the most common gynecologic disorders among young girls and is the major cause of activity restriction and college absence. A direct link between primary menstrual abnormalities and chronic conditions such as obesity has been established, though the exact pathology behind it is yet unknown.

Method

A total of 420 female students between 18 and 25 years of age from various professional colleges in a metro city were included in the study. Semi-structured questionnaire was used. Students were examined for height and weight.

Results

History of dysmenorrhea was given by 82.6% students. Out of these, 30% had severe pain and required medication. Only 20% took professional help for the same. There was a high prevalence of dysmenorrhea in participants who ate food outside frequently. Prevalence of irregular menstruation was more (41.94%) in girls having junk food three to four times a week.

Conclusion

The prevalence of dysmenorrhea and premenstrual symptoms were much higher as compared to the other menstrual abnormalities. The study revealed a direct association between consumption of junk food and an increase in dysmenorrhea.

## Introduction

Menstruation is a periodic and cyclical shedding of progestational endometrium accompanied by loss of blood [1]. Typically, a menstrual flow lasts two to seven days, and the duration between two menstrual cycles ranges from 21 to 45 days in 70-80% of cases. The most frequent menstrual disorders are dysmenorrhea, premenstrual syndrome, abnormal uterine bleeding, and amenorrhea, and they are individual-specific. These abnormalities of menstruation and variations in the menstrual pattern affect women’s physical, psychological, and social well-being. One-third to one-half of females with primary dysmenorrhea are missing school or work at least once per cycle, and more frequently 5% to 14% of them. The wide variation in the prevalence rates may be attributed to the use of selected groups of subjects [2]. Women particularly in India may feel shy and embarrassed to discuss their menstruation, and some stigma is associated with this subject in Indian culture; therefore, they hesitate to take medical help in case of menstrual problems. Dysmenorrhea is one of the most common gynecologic disorders among young girls and is the major cause of activity restriction and college absence. However, this condition is often considered as physiological pain and ignored. Problems associated with menstruation may lead to problems in academics and achievements in sports, as well as loss of self-image. Primary menstrual disorders as a health-related state havenumerous etiological factors such as obesity, hyperinsulinemia, and dyslipidemia, to name a few. A direct link between primary menstrual abnormalities and chronic conditions such as obesity has been established, though the exact pathology behind it is yet unknown [3]. Deviations in the menstrual patterns can stem from certain temporary causes such as mental and physical stress as well as chronic grievous causes such as hypogonadism, polycystic ovarian syndrome, and endometriosis, to name a few. Owing to the social stigma about menstrual abnormalities or menstruation in general, the multifactorial etiologies, and the varied variety of complications and debility caused due to it - ranging from missing schools/colleges to life-threatening conditions such as endometrial, ovarian, and cervical carcinomas - menstrual abnormalities are emerging as a “silent killer” as it is one of the most overlooked health-related state in our setup [4]. It is necessary to create awareness about the menstrual abnormalities as most of the factors leading to them are majorly modifiable. Therefore, the purpose of study was to find menstrual patterns and eating habits associated with it.

## Materials and methods

This is a cross-sectional study carried out over a period of two months. Female students in the age group of 18-25 years from various professional colleges in a metro city were included in the study. As per various studies, the prevalence of irregular menstrual cycle ranges from 17% to 33% [5]. Considering irregular menstrual cycle prevalence as 30%, the sample size of 323 was obtained using the formula 4pq/l2 with an allowable error of 5% (where p= prevalence, q=1-p, and l is the allowable error). However, all girls willing to participate in the study were included. Approval was taken from Dr. D. Y. Patil Medical College and Research Centre Institutional Ethics Sub Committee (number IESC/STS/2019/10). Purpose of the study was explained, and written informed consent was taken from the participants willing to participate in the study. Confidentiality was assured. A pre-tested semi-structured questionnaire was used. The questionnaire included items regarding the girls’ demographic details, as well as eating and drinking habits. The questions on the girls’ menstrual pattern concerned their age at menarche, duration of the most recent menstruation intervals, average days of bleeding, and any other menstrual problems and their frequency. The impact of the menstrual cycle on the girls’ physical and psychological health was also asked. Along with the questionnaire, students were examined for height and weight. Height was measured up to nearest centimeter using a measuring tape, and weight was measured up to the nearest kilogram using an electronic weighing scale. Data were entered in Microsoft Excel and analyzed using CDC Epi Info 7 software.

## Results

The mean age of students was 19.63 +1.72 years. A total of 420 students participated in the study, out of which 76% were MBBS (Bachelor of Medicine, Bachelor of Surgery) students, 20% were BDS (Bachelor of Dental Surgery) students, and the remaining 4% belonged to other health care professions. Majority (93.6%) of the participants were Hindu by religion. Around 62% participants had mixed diet. More than 80% stayed in a hostel, and 52% had increased frequency of eating outside. Increased frequency of eating junk food was seen in 50.7% participants. Body mass index (BMI) was calculated for 379 participants; out of these, 60.7% were normal and 27% were overweight. Participants were screened for pallor. Prevalence of anemia was found to be 22%. History of PCOD was positive in 12.8%, and 5.52% participants gave a positive history of having abnormal thyroid levels. Alcohol was consumed by 44.9%. Mean age of menarche was 12.96 + 1.48 years, with minimum age of 8 years and maximum age of 20 years. The average days of menstrual flow were observed to be in the range of four to six days in 64.44% of subjects. Most (60%) girls required three to five pads per day, and 25 % required change of pads during night.

History of dysmenorrhea was given by 82.6% students. Out of these, 30% had severe pain and required medication. Only 20% took professional help for the same. One-third of the respondents used over-the-counter medication. High incidence of premenstrual symptoms can be attributed to lack of physical activity and a relatively sedentary lifestyle of the healthcare students, as well as high intake of junk food and eating most of the meals outside. We also found out that eating outside food had a positive correlation with dysmenorrhea (using the chi-square test) (Table 1). We also found out that eating outside food had a positive correlation with dysmenorrhea. There was a high prevalence of dysmenorrhea in participants who ate food outside frequently (Table 1).

**Table 1:** Association of history of dysmenorrhea with junk food and eating outside

	Dysmenorrhea			
Frequency of junk food	Yes	No	Total	P-value
Daily	53 (91.38%)	5 (8.62%)	58	0.007
3-4 times	129 (82.17%)	28 (17.83%)	157	
Once a week	138 (83.64%)	27 (16.36%)	165	
Once a month	23 (63.88%)	13 (36.12%)	36	
Total	73 (17.55%)	343 (82.45%)	416	
Frequency of eating outside	Yes	No	Total	P-value
Daily	7 (10.29%)	61 (89.71%)	68	0.007
3-4 times	22 (14.77%)	127 (85.23%)	149	
Once a week	35 (19.55%)	144 (80.45%)	179	
Once a month	9 (16.67%)	13 (83.33%)	22	
Total	73 (17.46%)	345 (82.54%)	418	

Menstrual irregularity was seen in both underweight and overweight girls (23.40% and 29.41%, respectively); however, the chi square for trend was not significant. Prevalence of irregular menstruation was more (41.94%) in girls having junk food three to four times a week, as compared to those who were having junk food only once a week, where the prevalence was 21.29%.

## Discussion

In this study, the mean age of menarche was 12.96 years. Similar results were reported by the study conducted by Gumanga et al. [6]. The mean duration of menstruation flow was four days in the subjects of Dambhare et al. and three to eight days in the subjects of Sule and Ukwenya, which almost lies in concordance with our study [7,8]. Studies conducted in North America demonstrate early menarche with mean menarche age ranging from 12.02 to 12.16 years; 78.47% had an intermenstural duration of 21-35 days. Polycystic ovary syndrome affects approximately 5% to 10% of women in their reproductive life [9]. Around 25% girls had irregular menses and 82% had dysmenorrhea. In the study by Rigon et al., only 9% currently had irregular menses [10]. In the same study, the prevalence of dysmenorrhea was about 70%, of which only 6.3% were severe. The prevalence of dysmenorrhea in the study population of Negi. et al was 62.76% and in that of Dambhare et al. it was 56.1%, which was less than that our findings [11]. Majority of the participants gave a history of premenstrual symptoms. Regular physical exercise increases the levels of endorphins and encephalin that may help in decreasing the premenstrual symptoms and it may also allay anxiety and depression. Dambhare et al. in their study found that 65.5% of their subject gave a positive history of premenstrual symptoms [7]. Statistical significance was found between dysmenorrhea and intake of junk food. These results are in concordance with Negi et al [11]. Junk food is rich in saturated fatty acids and relatively deficient in micronutrients. The fatty acids may disrupt the metabolism of progesterone and lead to dysmenorrhea. Decreased micronutrient intake may potentiate this effect as most micronutrients act as cofactors for several enzymes that take part in metabolism. Incidence of dysmenorrhea was high in underweight girls as observed by Vani et al. [12]. Therefore, these findings suggest that undernourishment has an equally adverse effect on the menstrual cycle as over nourishment with respect to dysmenorrhea. Menstrual irregularity was seen in both underweight and overweight girls. These results are in concordance with the study conducted by Montero et al., who found that the attempt to lose weight causes irregular menstruation and dysmenorrhea [13]. Similar results were found in the study conducted by Fujiwara, where the degree of dysmenorrhea was greater in the girls who were dieting [14]. Prevalence of irregular menstruation was more (41.94%) in girls having junk food three to four times a week, as compared to those who were having junk food only once a week, where the prevalence was 21.29%. These results are in concordance with those of Negi et al. [11]. This irregularity can be attributed to the change in the hormonal profile due to subnormal progesterone levels that occur due to consumption of junk food.

Limitations

The junk food history given may not be reliable. Only the history of anemia and thyroid abnormalities was asked; thus, investigations could not be conducted for the same.

## Conclusions

Mean age of menarche was 12.96 +1.48 years, with minimum age of 8 years and maximum age of 20 years. The prevalence of dysmenorrhea and premenstrual symptoms were much higher as compared to the other menstrual abnormalities. The study reveals a direct association between consumption of junk food and an increase in dysmenorrhea. This finding opens up the potential prospect of conducting an interventional study, which will not only strengthen the aforementioned association but also help bring about a major change in the lifestyle of the population.
